# *In planta* Identification of Putative Pathogenicity Factors from the Chickpea Pathogen *Ascochyta rabiei* by *De novo* Transcriptome Sequencing Using RNA-Seq and Massive Analysis of cDNA Ends

**DOI:** 10.3389/fmicb.2015.01329

**Published:** 2015-12-01

**Authors:** Sara Fondevilla, Nicolas Krezdorn, Björn Rotter, Guenter Kahl, Peter Winter

**Affiliations:** ^1^Plant Molecular Biology, Institute for Molecular Bioscience, Goethe-University of FrankfurtFrankfurt am Main, Germany; ^2^GenXPro GmbHFrankfurt am Main, Germany

**Keywords:** MACE, RNA-Seq, *Ascochyta rabiei*, pathogenicity factors, transcriptome

## Abstract

The most important foliar diseases in legumes worldwide are ascochyta blights. Up to now, in the *Ascochyta*-legume pathosystem most studies focused on the identification of resistance genes in the host, while very little is known about the pathogenicity factors of the fungal pathogen. Moreover, available data were often obtained from fungi growing under artificial conditions. Therefore, in this study we aimed at the identification of the pathogenicity factors of *Ascochyta rabiei*, causing ascochyta blight in chickpea. To identify potential fungal pathogenicity factors, we employed RNA-seq and Massive Analysis of cDNA Ends (MACE) to produce comprehensive expression profiles of *A. rabiei* genes isolated either from the fungus growing in absence of its host or from fungi infecting chickpea leaves. We further provide a comprehensive *de novo* assembly of the *A. rabiei* transcriptome comprising 22,725 contigs with an average length of 1178 bp. Since pathogenicity factors are usually secreted, we predicted the *A. rabiei* secretome, yielding 550 putatively secreted proteins. MACE identified 596 transcripts that were up-regulated during infection. An analysis of these genes identified a collection of candidate pathogenicity factors and unraveled the pathogen's strategy for infecting its host.

## Introduction

Legume crops are a versatile and inexpensive source of protein for animal feeding and human consumption. Furthermore, legumes sustain agriculture by functioning as powerful natural soil fertilizers. They supply important added value to the crop by fixing atmospheric nitrogen, thus they reduce the need for artificial nitrogen fertilizer and help to minimize the adverse impact of agriculture on the environment. However, legumes are affected by many foliar and root diseases. The most important foliar diseases in legumes worldwide are ascochyta blights (Rubiales and Fondevilla, 2010).

Health or disease is the result of a battle between plants and their pathogens. Fungal pathogens use their “pathogenicity factors” to attack the plant, while recognition of the pathogen by the plant triggers defense responses. However, pathogens use effectors to suppress host immunity and some of these effectors induce host immunity upon recognition by the host's resistance gene(s) (Thakur et al., 2013). Therefore, plant resistance or susceptibility depends on the genetic background of both, pathogen and host. In the *Ascochyta*-legume pathosystem, most studies focused on the identification of resistance genes in the host (for a review see Rubiales and Fondevilla, 2012), while very little is known about the pathogenicity factors of *Ascochyta* spp. This study therefore aimed at the identification of pathogenicity factors of ascochyta blight pathogens using *Ascochyta rabiei* as a model.

*A. rabiei* (Pass.) Labrouse [teleomorph *Didymella rabiei* (Kovachevski) v. Arx] is a member of the dothideomycetes class of filamentous fungi. *A. rabiei* conidia germinate producing a germ tube and subsequently form an apressorium. Penetration occurs directly through the cuticle and some stomata. The penetration hyphae enter the plant tissue between two epidermal cells and then spread subepidermally inside the leaf and stem. Hyphae initially grow between epidermal and palisade parenchyma cells and proceed through the middle lamellae in parenchyma tissues from the leaflet through the petioles to the stem. This necrotrophic fungus kills host cells, producing necrotic lesions where pycnidia are formed (Ilarslan and Dolar, 2002; Jayakumar et al., 2005).

Very little is known about the pathogenic determinants of *A. rabiei*. For penetration of the host tissue and growth, *A. rabiei* produces cell-wall degrading enzymes such as a cutinase, xylanase and exopolygalacturonase (Tenhaken and Barz, 1991a; Tenhaken et al., 1997; Bruns, 1999). The pathogen also generates the phytotoxins solanapyrones A, B, and C, an unknown proteinaceous phytotoxin and Cytochalasin D (Höhl et al., 1991; Latif et al., 1993; Chen and Strange, 1994). *A. rabiei* is also able to degrade antimicrobial isoflavones and pterocarpan phytoalexins from chickpea (Kraft et al., 1987; Tenhaken et al., 1991b). Furthermore, the fungus produces a suppressor that inhibits the accumulation of antimicrobial compounds in chickpea (Kessmann and Barz, 1986). Although it has been demonstrated that the fungus generates these compounds in different media, it is unknown, whether it synthesizes them also during the infection process. Also, the role of these compounds in pathogenicity is unknown, except for the solanapyrone phytotoxin, which is not needed for pathogenesis, as mutants unable to produce the toxin did not show reduced virulence (Kim et al., 2013). Therefore, further studies are required to prove whether these already reported putative pathogenicity determinants are really induced during infection and contribute to pathogenicity.

Equally, little information about *A. rabiei* is available at the genomic level. Knowledge of the genome structure of *A. rabiei* is currently limited to a few genetic linkage maps containing few markers and a karyotype study (Lichtenzveig et al., 2002; Phan et al., 2003; Akamatsu et al., 2012). *A. rabiei* sequences are also limited since only 128 EST and 116 nucleotide sequences were available in NCBI database as per April 2015.

Next Generation Sequencing (NGS) enables the low-cost sequencing of whole genomes and transcriptomes and enormously increased the available sequence information for non-model organisms. Due to its unprecedented accuracy and specificity in quantifying differentially expressed genes, NGS also is a powerful tool for transcriptomic studies.

Here we used NGS for the *de novo* sequencing of the *A. rabiei* transcriptome, and to identify genes differentially expressed by the fungus during infection of chickpea leaves in comparison to the same fungus growing under artificial conditions.

## Material and methods

### Histological study to define the time course of the infection and select time sampling

A detailed histological study of the *A. rabiei*-chickpea pathosystem was performed prior to the start of the experiment to standardize the experimental conditions and define the timing of relevant steps of the *A. rabiei* infection process (spore germination, host's epidermis penetration, development of necrotic lesions in the host's mesophyll). Seeds from the susceptible chickpea cultivar “Blanco Lechoso” were individually sown in plastic pots containing 250 cm^3^ of a 1:1 sand-peat mixture. Plants were maintained at 20°C in a growth chamber with a 12 h light/12 h dark photoperiod until they reached the five-leaf stage.

Inoculations were performed on cut leaves placed into Petri dishes, in order to standardize the level of humidity, which greatly influences the speed of the development of the infection. For inoculation, the 3rd and 4th leaves of each plant were cut and placed on technical agar (4 g/l) in Petri dishes and inoculated by spraying them with a suspension of *A. rabiei* spores containing 12 million conidia/ml. The spore suspension was obtained by flooding the surface of 14 days old V8 cultures with sterile water, scraping the colony with a needle and filtering the suspension through two layers of sterile cheesecloth. The concentration of spores in the solution was determined with a haemocytometer. Finally, Tween-20 (120 μl per 100 ml of suspension) was added as a wetting agent. *A. rabiei* isolate P4, representing pathotype 4, was used for the experiments. This pathotype is the most virulent of the four described for the species, and chickpea accessions resistant to it are not available so far (Imtiaz et al., 2011). Isolate P4 originated from Kaljebrin, Syria and was kindly provided by Dr. Seid-Adhmed Kemal (ICARDA).

For histological studies samples were taken at 12, 24, 36, 48, 72, and 96 h after inoculation (hai). Cut leaflets were laid, adaxial surface up, on filter paper moistened with a 1:1 (v/v) mixture of glacial acetic acid: absolute ethanol for fixation and discoloration. To quantify the percentage of germinated spores, leaflets were mounted on lactoglycerol and stained by placing a drop of 5% aniline blue over them. For every leaf, the percentage of germinating spores was calculated by scoring in a light microscope each of 100 conidia for the presence of a germ tube. As this staining was not suited to reveal fungal structures within leaflets, the penetration into the epidermis and the formation of necrotic lesions in the mesophyll were quantified by first staining discolored leaflets by boiling in 0.05% tripan blue in a (1:2, v:v) lactophenol-ethanol mixture for 10 min. Leaflets were then moved into a solution of chloral hydrate (5:2, p:v) to clarify the tissues. One hundred germinated spores were examined and classified according to whether they had each formed a germ tube but not penetrated the epidermis, had penetrated the epidermis but not reached the mesophyll or had started to colonize the mesophyll and produce necrotic lesions. The experiment was replicated four times and for each histological variable one leaflet per replicate and time point was examined.

### Fungal material used for RNA extraction and sequencing

Total RNA was isolated from:

1. Mycelium of *A. rabiei* isolate P4 growing in the absence of its host (“*in medium*”): the fungi was grown for 1 week in PDB shaking media containing ampicillin and kanamycin (50 mg/ml each) in darkness at 20 ± 2°C. Two independent replicates were performed.
2. *A. rabiei* infecting chickpea leaves (“*in planta*”): leaves of the susceptible chickpea cultivar “Blanco Lechoso” were inoculated as described above for the histological study, and samples were taken at 12, 36, and 96 h after inoculation, corresponding to the following developmental stages: germination of spores, penetration of host epidermal cells and development of necrotic lesions in the host's mesophyll, respectively. Here, three replicates were performed.

RNA was extracted separately for each of the treatments and replicates using TRISure (Bioline, London, UK) according to the manufacturer's protocol. Integrity of total RNA was checked on agarose gels and its quantity and purity was determined using a NanoDrop ND1000 (NanoDrop Technologies, Inc., Wilmington, USA). Potential residual genomic DNA was removed using RQ1 RNase-Free Dnase (Promega, Madison, USA). RNA was further purified using the RNeasy Plant Mini Kit (Qiagen, Hilden, Germany).

### Library preparation and sequencing for RNA-Seq

For sequencing, the RNAs from the two replicates of the fungus grown “*in medium*” were mixed, while the RNAs from the tree replicates of the “*in planta*” treatment were sequenced separately. RNA-seq libraries were generated from 5 μg of total RNA by first capturing the mRNA using Oligo dT(25) beads (Dynabeads; life Technologies). The purified mRNA was then randomly fragmented in a Zn2+ solution and Illumina sequencing adapters were ligated to the RNA. cDNA was then generated using a p5 oligonucleotide for priming and the library was finally amplified with 10 cycles of PCR. Illumina HiSeq2500 (Illumina, Inc., San Diego, CA, USA) was used for paired-end sequencing (2 × 100 bp).

### Preparation and sequencing of massive analysis of cDNA ends (MACE) libraries

MACE libraries were produced for each replicate and treatment separately. The libraries were constructed from ≈5 μg of total RNA using the “MACE-Kit” (GenXPro GmbH, Frankfurt am Main, Germany) according to the supplier's manual. The technique analyzes 3′-biotinylated cDNA ends of 300–500 bp long, fragmented cDNA. These fragments are sequenced from their 5′-ends, thereby generating a single sequence from each transcript. In order to avoid bias during the PCR amplification steps included in the library preparation, GenXPro's PCR-bias-proof technology “TrueQuant,” implemented in the MACE kit, was employed allowing distinguishing PCR copies from original transcripts. For each library, 94-bp tags were sequenced on an Illumina HiSeq2500 machine (Illumina, Inc., San Diego, CA, USA).

### Preprocessing and mapping of illumina reads

The Illumina-derived sequence reads were processed with GenXPro's online in-house MACE analysis pipeline. Briefly, libraries were sorted according to their respective index, followed by elimination of duplicate PCR-derived tags identified by TrueQuant technology. Additionally, homopolymer filtering, quality filtering, removal of sequencing adapter primers and cDNA synthesis primers was performed. To distinguish *A. rabiei* reads from those originating from chickpea, reads derived from infected leaves were mapped against our own preliminary *A. rabiei* genome assembly (manuscript in preparation) and against the chickpea transcriptome reported by Hiremath et al. (2011) using the TopHat2.0.3 software (Trapnell et al., 2009). This draft *A. rabiei* genome assembly was obtained by sequencing *A. rabiei* isolate P4 using the Illumina platform. It consists of 913 scaffolds spanning 38.6 Mb with an N50 of 72 Mb. Reads mapping to the *A. rabiei* genome but not to the chickpea transcriptome were considered to originate from *A. rabiei* and selected for further analysis. In addition, to avoid any possible contaminant or artifacts, the sequences coming from the fungus growing in PDB medium were also mapped against the *A. rabiei* genome and only those that mapped there were used for the next steps.

### *A. rabiei* transcriptome sequencing and characterization

#### Transcriptome assembly

**Figure 1:**
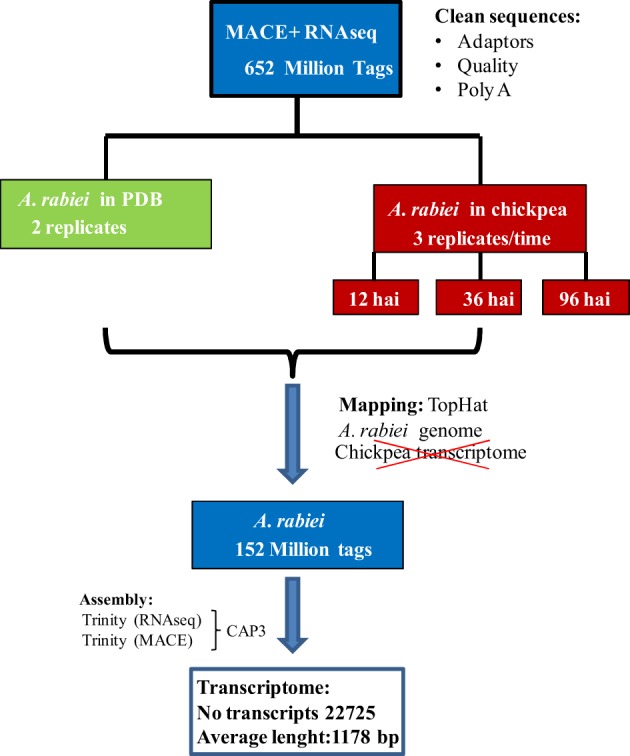
**Workflow followed for *de novo A. rabiei* transcriptome assembly**.

A summary of the workflow followed for the assembly of the *A. rabiei* transcriptome from RNA-Seq and MACE sequences is shown in Figure 1. RNA-Seq and MACE reads were first separately assembled using the Trinity software (Grabherr et al., 2011), indicating that the reads were strand-specific for improving the quality of the assembly. To reduce sequence redundancy, the scaffolds obtained from these separate assemblies were combined by the CAP3 software using default parameters (Huang and Madan, 1999). Both, the resulting contigs and singletons, were retained and sequences smaller than 200 bp were discarded. This set of sequences is called the *A. rabiei* transcriptome along the paper. To further remove redundant transcripts, singletons displaying >80% sequence similarity to CAP3-derived contigs were eliminated. This set of sequences is called the “reduced *A. rabiei* transcriptome.”

#### Transcript annotation

Putative functions were assigned to the resulting transcripts by mapping their *in-silico* translated protein sequences to the UniProtKB/Swiss-Prot, UniProtKB and RefSeq_protein databases using the BlastX algorithm available at NCBI (http://www.ncbi.nlm.nih.gov) in hierarchical manner, using an *e*-value of 10^−5^ as a threshold for considering them as homologs. Transcripts without homology to these sequences were subsequently annotated to the non-redundant nucleotide NCBI database (nr) by BLASTN using an *e*-value of 0.001 as a threshold. In order to favor informative hits, modified UniProtKB/Swiss-Prot, UniProtKB, RefSeq_protein, and nr databases were used for the BLAST. These modified databases contained only entries originating from fungi. In addition, entries containing the terms “uncharacterized protein” and “predicted protein” were removed from UniProtKB database, those containing the terms “chromosome,” “genome,” “clone,” “cosmid,” and “YAC,” were removed from the nr (nucleotide) database and entries containing “hypothetical protein” were remove from all databases. Transcripts without a hit in these modified databases were blasted as reported above against all unmodified databases.

Conserved protein families/domains were identified by translating the transcripts into protein using an in-house script and searching against the Pfam protein family database (Finn et al., 2014) with the PfamScan software (Mistry et al., 2007).

For classifying the transcripts into functional categories, GO-terms were assigned to transcripts according to their blast UniProtKB hits and classified to GO Slim terms using the tool GoSlimViewer (McCarthy et al., 2006).

#### Prediction of secreted proteins

The predicted amino acid sequences of *A. rabiei* transcriptome were searched for putative secreted proteins. Sequences harboring a signal peptide cleavage site at the N-terminal region according to SignalP4.1 (Petersen et al., 2011) but lacking a transmembrane region as predicted by TMHMM2.0 (Krogh et al., 2001) were considered putatively secreted proteins.

### Identification of transcripts differentially expressed during host infection

**Figure 2:**
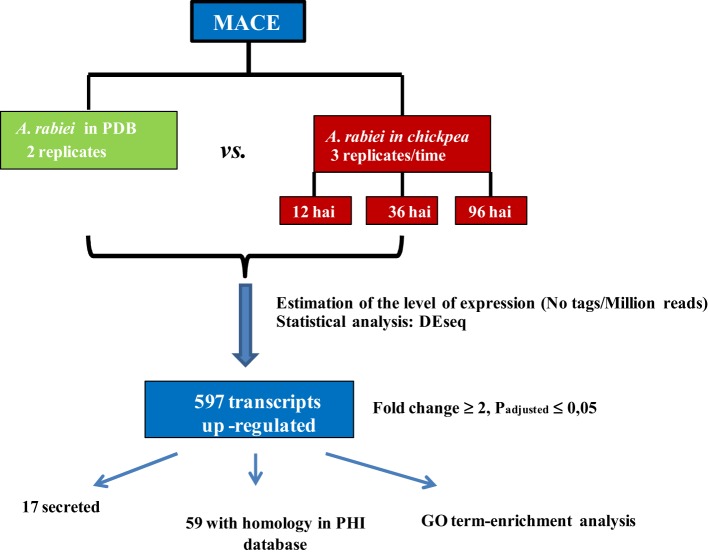
**Workflow followed to identify transcripts up-regulated “*in planta*” *vs*. “in medium” treatments**.

A summary of the workflow employed for identification of transcripts differentially expressed during the infection process is shown in Figure 2. The reduced *A. rabiei* transcriptome was used for assignment of the transcripts. Transcript frequencies were calculated using a custom workflow. Briefly, MACE reads were mapped to the reduced *A. rabiei* transcriptome using the SOAP2.2 software (Li et al., 2008) allowing five base mismatches. Normalization and test for differential expression were performed using the DEseq package (Anders and Huber, 2010). To identify transcripts over-expressed during infection, normalized expression values were compared between “*in planta*” libraries and “*in medium*” libraries. A transcript was considered to be up-regulated during infection when the normalized expression value of the transcript was statistically significantly different (p_adjusted_ ≤ 0.05), and at least two times higher, “*in planta*” compared to “*in medium*” treatment for at least one time point after inoculation, or when the transcript was only sequenced in “*in planta*” libraries. As the number of transcripts from the fungus in “*in planta*” libraries were markedly smaller than “*in medium*” libraries, the absence or lower frequency of a transcript “*in planta*” compared to “*in medium*” was not considered to be accurate enough to define a transcript as down-regulated during infection. Therefore, only transcripts over-expressed “*in planta*” are reported in this study.

To develop a heat map showing the expression pattern of the up-regulated transcripts at the different time points after inoculation, for each time point, a value of “1” was assigned to up-regulated transcripts, a value of “−1” to those transcripts that were only sequenced in “*in medium*” libraries and a value of “0” to the remaining cases. A matrix containing these values was submitted to cluster analysis by TIGR Multiexperiment Viewer (MeV) 4.8 software. Using Pearson's correlation distance method, data were clustered employing the average linkage clustering routine under hierarchical clustering.

To predict potential pathogenicity factors of *A. rabiei*, those genes over-expressed during infection were BLASTed against the pathogen-host interaction gene database (PHI database) using the BlastX algorithm available at NCBI and an *e*-value of 10^−5^ as a threshold. The PHI database (Winnenburg et al., 2006) is a collection of experimentally verified pathogenicity, virulence, lethal, and effector genes from fungi and bacteria.

Using the BLASTN algorithm we additionally identified the sequences corresponding to some already known *A. rabiei* genes in our transcriptome, and examined whether they were up-regulated during infection. These genes encoded a cutinase (Tenhaken et al., 1997), the polyketide synthases *pks1* and *pks2*, the putative *A. rabiei* virulence factor *cps1* (White and Chen, 2007; Akamatsu et al., 2010), or were induced after oxidative stress (Singh et al., 2012).

#### Go-enrichment analysis

To determine which GO Slim terms were over- or under-represented in the subset of predicted secreted proteins, or in the subset of genes stronger expressed during infection, χ^2^ statistics was used to compare the percentage of transcript belonging to each GO Slim category between these subsets of transcripts and the whole transcriptome.

### Validation of expression profiles of putative pathogenicity factor genes by qRT-PCR

The expression profiles of eleven *A. rabiei* genes that, according to MACE, were stronger expressed “*in planta*” than “*in medium*,” were examined with two-step qRT-PCR.

Polymerase chain reactions were performed in a 48-well plate with a StepOne Real Time PCR System (Applied Biosystems, Foster City, CA, USA), using EvaGreen to monitor dsDNA synthesis. Reactions contained 2.4 μl of 5 × HOT MOLPol EvaGreen qPCR mix (ROX), 7 μl of cDNA, and 0.08 μM of each gene-specific primer in a final volume of 12 μl. The following standard thermal profile was used for all PCR reactions: polymerase activation (95°C for 15 min), amplification and quantification (40 cycles; 95°C for 15 s, 60°C for 1 min) and dissociation curve generation (95°C 15 s, 60°C 1 min, 95°C 15 s). Primers were designed using Probe Finder 2.45 (Universal Probe Library, Roche) or with Oligo 6 primer analysis software (Molecular Biology Insights, Inc). The absence of a target in the chickpea genome and transcriptome was checked. Selected genes and corresponding primer sequences are listed in Table 1. The genes “γ-tubulin complex component GCP6” and “α-actinin-2” genes were used as references for normalization. These genes were not differentially regulated in any treatment according to MACE results.

**Table 1:** **Primer sequences for the amplification by qRT-PCR**.

**Trancript**	**Annotation**	**Forward primer (5′-3′)**	**Reverse primer (5′-3′)**
Contig7924	Gamma-tubulin complex component GCP6	agaaaggatatgggcggaag	gcaactaccagcctctcgat
Contig5750	Alpha-actinin-2	cggcgtcagacttggataac	aaatacccagtgccccctat
Contig13264	Probable alpha-N-arabinofuranosidase B	tggtgttcatggcttcgac	gattgcataacgatgtgcctaa
Contig12940	Cuticle-degrading protease	catcttgacaatgtattttccg	acctgttcccgttgctga
Contig11798	Pisatin demethylase	tcgggaagaacataagcctct	aaatcgaatttgcgaacaatct
Comp79036_c0_seq1	Integral membrane protein (Pth11)	tcggtgagatgatgatcgtg	gaggggaggttgtgggtaat
Comp7688_c2_seq1	MAP kinase kinase skh1/pek1	ctcgaagtcgcacaacatcg	taggaattggttgtcgaacaat
Comp56538_c0_seq1	Isotrichodermin C-15 hydroxylase	ctttgggtgtttggaaaacg	cataaatgttcgcaaagacgag
Contig11477	OsmC family protein	tttaatcaagcattccccaca	aaaggcgagtggtggtaagtt
Comp6720_c1_seq1	Fungal specific transcription factor domain containing protein	aacgtgtgctctcattcagc	tgcagctcgtgaatactcca
Contig6518	Non-histone chromosomal protein 6A	gagcttcaacaacgactaccg	agccgatcaggggtacaag
Contig13025	Phospholipase D/Transphosphatidylase	aggcgaggagctgtcatatc	gttgccgttaccttggattc
Comp427_c0_seq1	Probable rhamnogalacturonate lyase A	gcatctggtggtccgttc	agatatcgtaaaggtcaggtcca

The PCR efficiency of each primer pair in each individual reaction was calculated using LingRegPCR 7.5 software and used to calculate an average efficiency (E) per primer pair. This average efficiency was used to calculate the expression in each reaction using the formula Expresion = E^CT^. A normalization index was calculated for each plate as the geometric mean of the expression of the reference genes “γ-tubulin complex component GCP6” and “α-actinin-2” and a relative expression was calculated for each reaction as the ratio of the gene expression of the gene of interest in each reaction against the normalization index.

### Identification of gene clusters involved in toxin metabolism

In order to identify in *A. rabiei* orthologues of the genes involved in the metabolism of the toxins solanapyrone, aflatoxin, and tricothecene and the presence of potential gene clusters controlling their synthesis, we annotated our *A. rabiei* trancriptome assembly by BlastX (*e* = 10^−4^) against the proteins corresponding to the solanapyrone cluster in *Alternaria solani* (Kasahara et al., 2010), the aflatoxin biosynthesis gene cluster in *Aspergillus parasiticus* (Yu et al., 2004) and the tricothecene biosynthesis genes in *Fusarium sporotrichioides* (accessions gb|AF359360.3, gb|AY040587.1, gb|AY187275.1, and gb|AF127176.1). The position of the respective homologous *A. rabiei* transcripts in the *A. rabiei* genome was estimated by blasting the respective contigs against our own *A. rabiei* genome assembly (BLASTN, *e* = 10^−4^).

## Results

### *De novo A. rabiei* transcriptome assembly

In order to obtain as many different transcripts as possible, we sequenced cDNA libraries from the fungus growing in four different conditions. These included the fungus growing in a liquid medium in the absence of its host and the fungus infecting chickpea leaves at three relevant steps of the infection process: germination of conidia, penetration of host epidermal cells and development of necrotic lesions in the host's mesophyll. To define the sampling time points for these steps, we performed a histological study under the same conditions as those used for sampling the material for sequencing. A time course of these infection steps is shown in Additional file 1. According to this time course, 12, 36, and 96 hai were considered to represent germination, penetration of the epidermis and starting of appearance of necrotic lesions in the host's mesophyll. At 12 hai *A. rabiei* germination had started but penetration of host's epidermal cells was not observed yet. At 36 hai, germination had finished and a good proportion of spores were penetrating the chickpea epidermis. At 96 hai penetration had finished and necrotic lesions appeared in the mesophyll. At this time point some lesions were already visible macroscopically on chickpea leaves. Miscroscopic images illustrating these *A. rabiei* developmental steps are shown in Additional file 2. cDNA libraries from the different treatments were sequenced using RNA-Seq and MACE. After elimination of duplicates and quality trimming a total of 309,296,583 and 342,856,274 sequencing reads were processed for RNA-Seq and MACE, respectively (Table 2). Of these 86,463,866 RNA-Seq and 65,701,408 MACE sequences belonged to *A. rabiei*. The percentage of *A. rabiei* sequences in “*in planta*” samples was 0.73 on average in RNA-Seq and 2.38 in MACE experiments.

**Table 2:** **Summary of *A. rabiei* reads counts**.

	**RNAseq**			**MACE**		
	**Reads after filtering**	**Read mapped to *A. rabiei* genome**	**% reads from *A. rabiei***	**Reads after filtering**	**Read mapped to *A. rabiei* genome**	**% reads from *A. rabiei***
“In medium”	133,272,362	85,179,642	63.91	67,073,871	59,134,006	88.16
“ *In planta*”	176,024,231	1,284,224	0.73	275,782,403	6,567,402	2.38
Total	309,296,593	86,463,866		342,856,274	65,701,408	

For the *de novo* assembly of the *A. rabiei* transcriptome, single-end MACE reads and paired-end RNA-Seq reads were first assembled separately. For the assembly we used Trinity, as this software distinguishes between different isoforms. The assembly of MACE reads with Trinity yielded 28,373 contigs with an average length of 460 bp, while the assembly of RNA-Seq reads resulted in 31,830 contigs with an average length of 932 bp. To reduce redundancies, MACE and RNA-Seq contigs were merged using CAP3. The resulting *A. rabiei* transcriptome contains 22,725 transcripts with an average length of 1178 bp and is provided in Additional file 3. To further eliminate over-represented transcripts, those singletons having a sequence similarity with CAP3-derived contigs >80% were eliminated. This reduced transcriptome contained 20,639 transcripts with an average length of 1172 bp and is provided in Additional file 4.

### Characterization of the *A. rabiei* transcriptome

**Figure 3:**
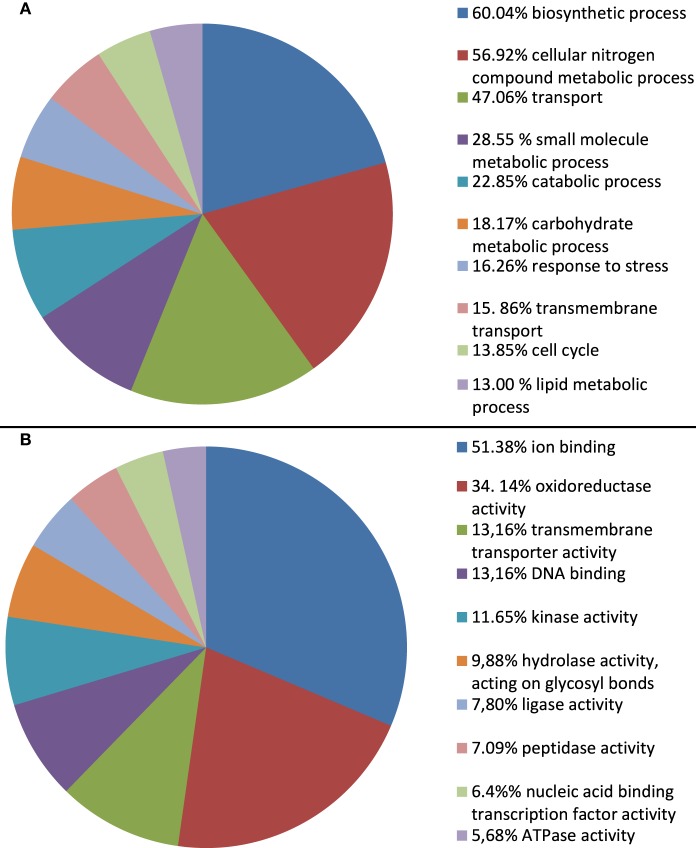
**Percentage of *A. rabiei* transcripts belonging to each GO Slim term for the GO categories (A) Biological process and (B) Molecular function**. Only the 10 most frequent GO Slim terms are shown.

To infer the putative functions of *A. rabiei* transcripts we used BLAST. In order to favor informative hits, modified UniProtKB/Swiss-Prot, UniProtKB, RefSeq_protein, and nr databases, lacking uncharacterized protein or nucleotide sequences were used for BLASTing. Out of 22,725 transcripts representing the *A. rabiei* transcriptome, 14,116 could be annotated using this approach. Of these, 8421 matched to entries in the UniProtKB/Swiss-Prot data base, 5285 to UniProtKB and 410 to RefSeq_protein and nr database entries. The associated hits were searched for their respective Gene Ontology (GO) terms and classified into GO Slim terms using GoSlimViewer software. For the GO category “Biological process” the most represented GO Slim terms were “biosynthetic process,” “cellular nitrogen compounds metabolic process,” “transport,” “small molecule metabolic process,” and “catabolic process” (Figure 3). For the GO category “Molecular function,” the most abundant GO Slim terms were “ion binding,” “oxidoreductase activity,” “transmembrane transporter activity,” “DNA binding,” “kinase activity,” and “hydrolase activity, acting on glycosyl bonds” (Figure 3).

**Figure 4:**
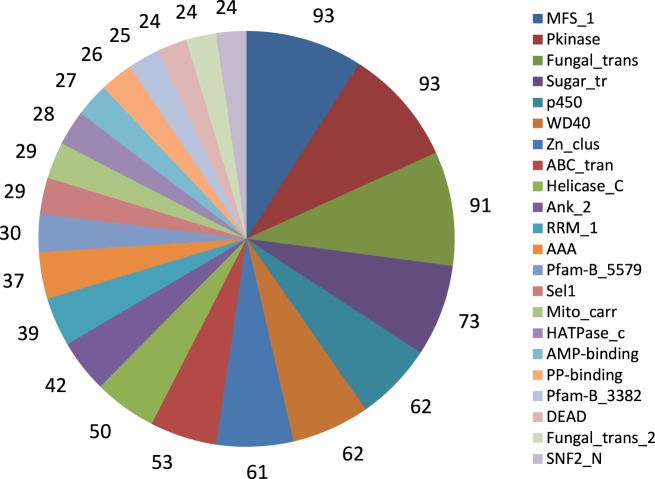
**Twenty-two frequent Pfam protein families found in *A. rabiei* transcriptome**. The number of proteins included in the respective family is indicated.

We further identified conserved protein families/domains present in the *A. rabiei* transcriptome by searching against Pfam protein domain families. The top 22 more frequent families/domains are shown in Figure 4. The frequent families/domains in the *A. rabiei* transcriptome were related to transport and regulation of genes such as “Major facilitator superfamily,” “Protein kinase domain,” “Fungal specific transcription factor domain,” “Sugar (and other) transporter,” “WD domain; G-beta repeat,” “Fungal Zn(2)-Cys(6) binuclear cluster domain,” and “ABC transporter.” Other relevant domains were associated with toxin/secondary metabolism such as “Fungal thricothecene efflux pump TRI12” (eight proteins), that are involved in tricothecene toxin biosynthesis, “Polyketide synthase” (12 proteins), the “KR domain,” found in polyketide synthase toxin biosynthesis (eight proteins), and different kinds of “Beta-ketoacyl synthase,” many of which are also involved in the synthesis of polyketides (19 proteins). Many protein domains with putative roles in plant tissue degradation were also identified as e.g., different kinds of peptidases domains (79 proteins), “pectinesterases” (three proteins), “pectate lyase” (11 proteins), “lipases” (11 proteins), “Glycosyl hydrolases” (127 proteins), “cutinase” (five proteins) and “cellulase” (four proteins).

Some Pfam domains found in *A. rabiei* were domains only present in phytopathogens such as PF02182 (YDG/SRA domain), PF02682 (Allophanate hydrolase subunit 1), and PF06916 [Protein of unknown function (DUF1279)] (Soanes et al., 2008). Other domains such as PF07110 (EthD protein), PF00024 (PAN domain), PF02705 (K+ potassium transporter), PF03572 (Peptidase family S41), and PF01083 (Cutinase) were found in *A. rabiei* in a higher proportion than on average in non-pathogenic filamentous ascomycetes (Soanes et al., 2008).

### The *A. rabiei* secretome

**Figure 5:**
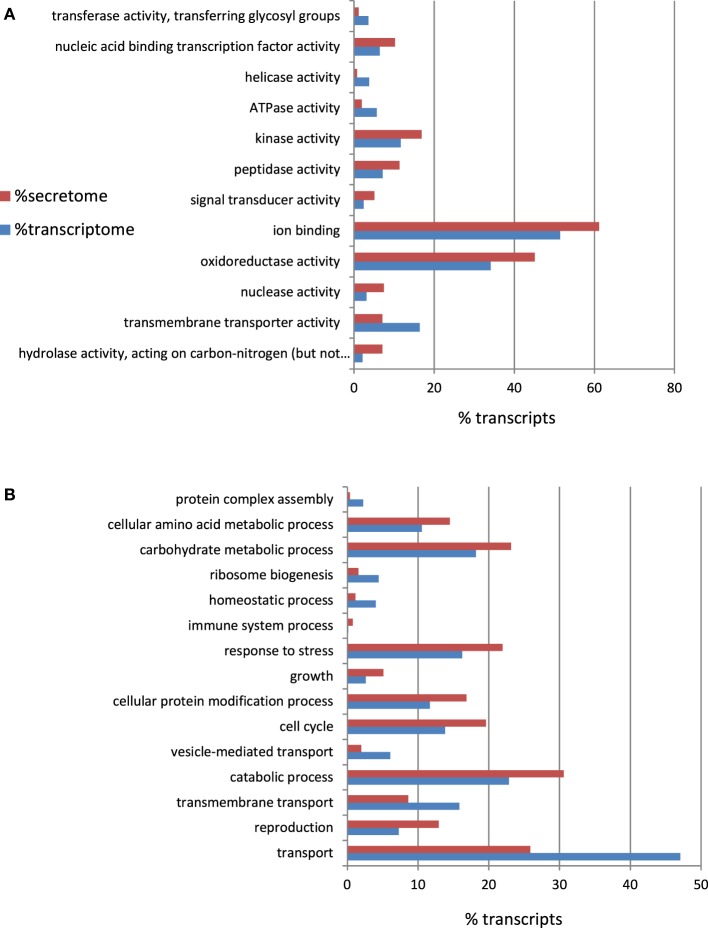
**Percentage of *A. rabiei* transcripts belonging to over/under-represented (χ2, *p* < 0.05) GO Slim terms in the predicted secretome in comparison to the complete transcriptome for the GO categories (A) Molecular Function (B) Biological process**.

To a large extent, the interaction between a pathogen and its host is orchestrated by the proteins that are secreted by the pathogen into the host (Hane et al., 2007). Therefore, *in-silico*-translated amino acid sequences from the *A. rabiei* transcriptome were searched for putatively secreted proteins. Sequences with a signal peptide but no transmembrane region were considered as candidates. A total of 550 proteins were predicted to be secreted (see Additional file 5). These included degrading enzymes, such as peptidases, hydrolases, glucosidases, and proteases. Indeed, as depicted in the GO Slim enrichment analysis (Figure 5), the secretome of *A. rabiei* was enriched in proteins belonging to the “Molecular function” GO categories “hydrolase activity, acting on carbon-nitrogen (but not peptide) bonds” and “peptidase activity.” Other Go-Slim terms over-represented in the secretome were “nucleic acid binding transcription factor activity,” “kinase activity,” “ion binding,” and “oxireductase activity. Interestingly, the GO biological processes “response to stress” and “catabolic process” were over-represented in the secretome in comparison to the whole transcriptome. Other GO biological processes over-represented in the secretome were “cellular amino acid metabolic process,” “carbohydrate metabolic process,” “growth,” “cellular protein modification process,” “cell cycle,” and “reproduction”.

### Identification of genes differentially expressed during host infection

For quantification of gene expression we selected MACE, a versatile, digital transcriptome profiling method. Contrary to RNA-Seq (Nagalakshmi et al., 2008), in which for every transcript several sequences may be generated, in MACE each cDNA molecule is represented by one sequence (the tag) of 94 bp, originating from a region around 100–500 bp from the 3′ (poly-A) end of the transcript. High-throughput sequencing of tags provides numerical gene expression values and reveals the expression of even lowly abundant transcripts beyond the scope of microarrays and RNA-Seq, if sequenced at similar depth (Zawada et al., 2014; Nold-Petry et al., 2015). Thus, MACE is ideally suited to identify even rare fungal transcripts in a high background of plant mRNAs as e.g., in leaves of chickpea early after infection. Since after assembly of the tags, MACE sequence assemblies comprise only around 500 bp from each transcript species, we performed full-length RNA-Seq to obtain longer sequence assemblies that could be used as a reference for the annotation of the MACE tags.

**Figure 6:**
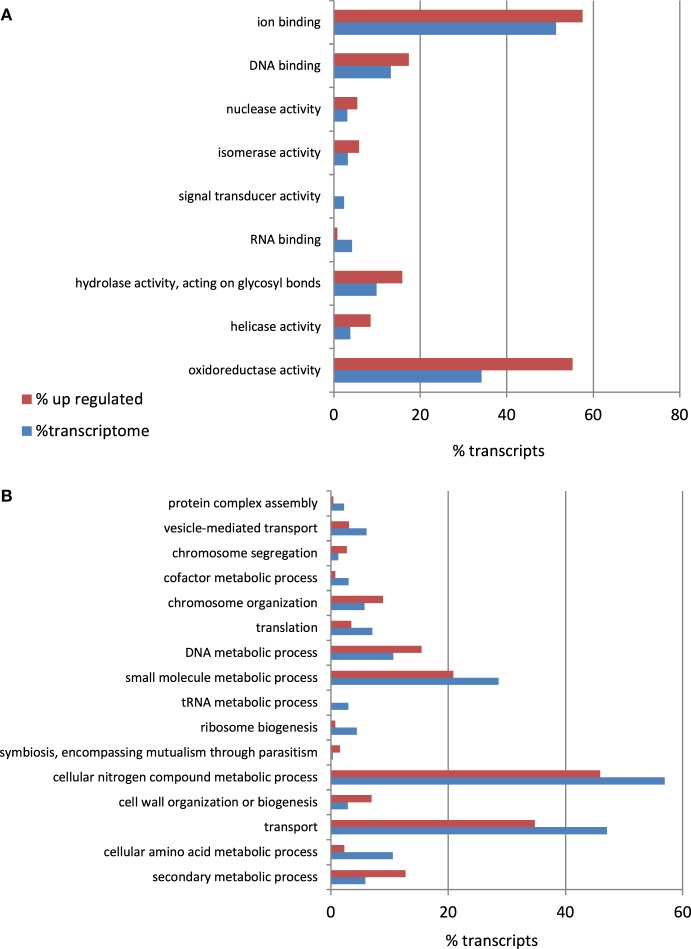
**Percentage of *A. rabiei* transcripts belonging to over/under-represented (χ2, *p* < 0.05) GO Slim terms in the set of up-regulated genes in comparison to the complete transcriptome for the GO categories (A) Molecular Function (B) Biological process**.

To identify the *A. rabiei* genes specifically involved in host infection, we compared the gene expression profiles of the “*in medium*” with the “*in planta*” growing pathogen at three relevant steps of the infection process. Statistical analysis of the transcriptome data with DEseq identified *A. rabiei* genes consistently differentially expressed during the infection process across three independent replicates. In total 596 transcripts were up-regulated at least at one time point after infection in comparison to the fungus grown in medium (Additional file 6). The largest number of up-regulated transcripts (431) was up-regulated at the earliest steps of infection at 12 hai, while 353 and 342 were up-regulated at 36 and 96 hai, respectively. A total of 179 transcripts were up-regulated under all infection conditions. A heat map showing nine clusters describing the expression pattern of these up-regulated transcripts at the different time points is shown in Additional file 7. The transcripts included in each cluster can be found in Additional file 8. GO term enrichment analysis revealed that genes categorized to the GO biological processes “secondary metabolic process,” “cell wall organization and biogenesis,” “symbiosis, encompassing mutualism through parasitism,” “DNA metabolic process,” “Chromosome organization,” and “Chromosome segregation” were overrepresented in up-regulated genes compared to the whole transcriptome (Figure 6). For the GO category “Molecular function,” an enrichment of genes showing “oxyreductase activity,” “helicase activity,” “hydrolase activity, acting on glycosyl bonds,” “isomerase activity,” “nuclease activity,” “DNA binding,” and “ion binding” was detected.

A total of 59 transcripts with homology to genes included in the PHI database and known to be involved in pathogenicity in other pathogenic fungi were more expressed during infection (Additional file 6). The transcripts corresponding to two *A. rabiei* genes previously identified in response to oxidative stress (*Ar125, Ar126*) and the polyketide synthase *pks2* were also found to be over-expressed during infection, but not *pks1* and *cps1* (Tenhaken et al., 1997; White and Chen, 2007; Akamatsu et al., 2010; Singh et al., 2012).

Pathogenicity factors are usually secreted by the fungus into the host, and 17 up-regulated transcripts encoded predicted secreted proteins. Genes potentially relevant for *A. rabiei* pathogenicity that we identified comprise genes for cell wall-degrading enzymes, for toxin metabolism and for enzymes for detoxification of fungitoxic compounds produced by the plant as defense (Additional file 9). qRT-PCR confirmed the MACE results, as 10 out of 11 genes checked revealed the same pattern of expression with both techniques (Table 3).

**Table 3:** **Comparison of expression values (average expression ratio inoculated/control) obtained by MACE and qRT-PCR**.

**Transcript**	**Annotation**	**MACE**	**qRT-PCR**
Contig13264	Probable alpha-N-arabinofuranosidase B	171.91	168.41
Contig12940	Cuticle-degrading protease	2478.24	497.65
Contig11798	Pisatin demethylase	145.46	150.74
Comp79036_c0_seq1	Integral membrane protein (Pth11)	Only “*in planta*”	1.99
Comp7688_c2_seq1	MAP kinase kinase skh1/pek1	97.74	0.56
Comp56538_c0_seq1	Isotrichodermin C-15 hydroxylase	Only “*in planta*”	720.68
Contig11477	OsmC family protein	58.85	36.82
Comp6720_c1_seq1	Fungal specific transcription factor domain containing protein	Only “*in planta*”	4.95
Contig6518	Non-histone chromosomal protein 6A	163.25	6.35
Contig13025	Phospholipase D/Transphosphatidylase	347.67	69.96
Comp427_c0_seq1	Probable rhamnogalacturonate lyase A	Only “*in planta*”	8.95

### Identification of toxin gene clusters

*A. rabiei* is known to produce the toxins solanapyrone A, B, and C (Höhl et al., 1991). In our study also several transcripts that were up-regulated during infection showed homology to genes involved in aflatoxin and tricothecene toxin metabolism from other fungi. In their genomes these toxin synthesis genes are clustered. Therefore, we searched in *A. rabiei* for orthologs of the genes involved in the synthesis of these toxins and their corresponding gene clusters.

*A. rabiei* transcripts for proteins with high identity (>90%) to those encoded by genes located on the solanapyrone biosynthesis gene cluster of *A. solani* (Kasahara et al., 2010) were identified for all the genes of the cluster except *sol3* (Additional file 10). These genes were also clustered in the *A. rabiei* genome (manuscript in preparation). Therefore, we could identify the *A. rabiei* genes belonging to this cluster unambiguously. None of these genes were up-regulated during infection.

Contrary to the solanapyrone gene cluster, we could not identify clustering of aflatoxin and tricothecene biosynthesis genes in the *A. rabiei* draft genome. Although many genes from *A. rabiei* with homology to aflatoxin and tricothecene biosynthesis genes were identified, their translated proteins were only moderately identical (< 50% identity) to the respective genes from *A. parasiticus* and *F. sporotrichioides*, where these genes do form clusters. Thus, currently neither their genomic position nor their sequence identity allows the unambiguous identification of orthologs of these genes in *A. rabiei*.

## Discussion

Despite the high relevance of *Ascochyta* spp. as plant pathogens, very little was known until now about their pathogenicity factors and only a few molecular sequences were available for these species. This scenario has now changed drastically with the present paper in which we report a comprehensive *A. rabiei* transcriptome and identify several putative pathogenicity factors specifically induced during infection.

This detailed analysis was possible due to the high sequencing depth and the accuracy of our gene expression measurement. Studying the gene expression profile of pathogens during host infection is not an easy task. As only a small number of plant cells are invaded by the pathogen, the proportion of fungal cells compared to plant cells is very low, especially in the early phases of infection. This makes it difficult to detect fungal transcripts that are specifically expressed during infection in the mixed RNA from plant and pathogen and, especially, to retrieve a number of fungal sequences sufficient to perform an accurate expression analysis across replicates. This was achieved in our study where we exploited the advantages of MACE to produce precise genome-wide transcription profiles even in the infected tissue at reasonable costs. Up to 6,567,402 MACE sequences from the fungus representing the same number of transcripts were obtained from the infected tissue. This corresponds to around 2.4% of the total number of sequences from the “*in planta*” materials. An additional 1,284,224 fungal sequences (0.73% of the total sequencing reads), were recovered by sequencing RNA from the infected tissue with RNA-Seq. All together, we analyzed more than 152 million sequences from “*in medium*” and “*in planta*” material. This high coverage, together with a restrictive statistical analysis, allowed us the identification of genes consistently differentially expressed during the early infection process across three independent replicates.

Due to the depth of our analysis we predict the *A. rabiei* transcriptome to contain between 20,639 to 22,725 transcripts. The mean contig length of the assembled transcriptome was around 1172 bp. This length fits well to the length of transcripts expected for Dothideomycetes (Rouxel et al., 2011), highlighting the good quality of the transcriptome. This transcriptome will clearly enlarge the genomic resources available for *A. rabiei* and will be a useful repository to identify genes of interest in this and related species. As an example, we identified the transcripts from the solanapyrone biosynthesis gene cluster from *A. rabiei*. We further predicted the *A. rabiei* secretome comprising 550 putatively secreted proteins. Such proteins are crucial for successful colonization of the host by phytopathogens, since they depolymerize plant cell constituents, kill the cells or suppress the host's defense.

We here not only report a collection of genes which - according to their annotation—may have a putative role in pathogenesis but, more interestingly, identify which of them are specifically induced or up-regulated during infection. Moreover, we could predict which of these infection-specific genes may be secreted as additional evidence for their importance for pathogenesis. In the following we will discuss a collection of putative pathogenicity factors that we identified, that could be major constituents of the fungal war machinery.

### Plant tissue degrading enzymes

Our results show that *A. rabiei* produces a battery of degrading enzymes, probably needed to penetrate and colonize its host. The first barrier for the pathogen to infect its host is the cuticle. Aerial plant organs are protected by a cuticle composed of i.a. the insoluble polymere cutin, a hydroxyl, and hydroxyepoxy fatty acid polyester (Ettinger et al., 1987). Until now only a cutinase, encoded by the *cut* gene, has been reported for *A. rabiei* (Tenhaken et al., 1997). Besides this gene (corresponding to MACE_comp207687_c0_seq1) we identified five additional transcripts for proteins harboring a cutinase domain. The *cut* gene transcript was not differentially expressed during infection in our study. However, a “cuticle degrading protease” transcript was up-regulated during infection and two cutinase transcripts were only expressed in the “*in planta*” material and could therefore be important for the first step of infection.

After crossing the cuticle barrier, plant pathogens need to breach the host cell-wall, and therefore produce degrading enzymes such as cellulases, polygalacturonases, xylanases, proteinases (Annis and Goodwin, 1997), and others to macerate the tissue and produce soft rot. An arsenal of genes encoding degrading enzymes where over-expressed or only expressed during infection in our study (Additional files 6 and 9). The only known *A. rabiei* xylanase (encoded by the *abieixyl1* gene), was not over-expressed during infection in our experiment. Instead we identified two transcripts for “1,4-beta-xylosidase,” an enzyme involved in xylan hydrolysis.

Furthermore, one GO functional categories most represented in the *A rabiei* transcriptome was “hydrolase activity, acting on glycosyl bonds.” This functional category was also over-represented in the subsets of mRNAs up-regulated during infection and in the secretome (Figures 3, 5, 6). Glycoside hydrolases that participate in the degradation of host cell walls also play a role in degrading the fungal cell wall during cell division, growth and morphogenesis (Kawahara et al., 2012), crucial processes for developing the specific fungal structures needed to colonize the host.

“Peptidase” was another over-represented functional category in the subset of *A. rabiei* secreted proteins. Secreted pectic enzymes cleave internal glycosidic bonds in structurally important pectin polymers in the middle lamella and primary cell walls of plant tissues, resulting in tissue maceration and plant cell death (Schneider and Collmer, 2010). Genes encoding enzymes belonging to this group and over-expressed during infection were also identified in our study.

### Protection against fungitoxic compounds

To survive in its host the pathogen needs to counteract the toxic compounds produced by the plant as a defense. These include reactive oxygen species (ROS) and fungitoxic secondary metabolites such as phenylpropanoids, terpenoids, phytoalexins, and glucosinolates. Oxidoreductases detoxify ROS and thereby protect the pathogen against the aggressive environment created by the plant. Accordingly, “Oxydoreductase activity” was one of the most enriched GO functional categories in the *A. rabiei* transcriptome and was also enriched in the subset of genes up-regulated during infection and in the secretome. A syntenic block of genes including oxidoreductases is conserved in most Dothideomycetes and also up-regulated during infection in *Lepstosphaeria maculans* (Ohm et al., 2012). As additional evidence for their protective role against ROS in the *A. rabiei*-chickpea compatible interaction, two of the transcripts over-expressed during infection, Contig464 (NADH-ubiquinone oxidoreductase chain 6), and Contig8395, corresponded to two *A. rabiei* genes, *Ar125*, and *Ar126* respectively, identified in response to oxidative stress before by Singh et al. (2012).

Phytoalexins are low molecular weight antimicrobial compounds produced by plants in response to pathogen attack. Several fungal plant pathogens are able to detoxify phytoalexins and numerous studies have demonstrated a link between the ability to detoxify phytoalexins and pathogenicity (Enkerli et al., 1998). Our results suggest that the detoxification of phytoalexins produced by chickpea also plays a role in *A. rabiei* pathogenicity as the predicted protein of one of the transcripts expressed only during infection (comp21970_c0_seq1) share high sequence identity (49%) with that encoded by the *MAK1* gene that degrades the chickpea phytoalexin maackiain (Enkerli et al., 1998). Furthermore, two other up-regulated transcripts coded for “pisatin demethylase.” Though pisatin is the predominant phytoalexin of pea, the presence of two related transcripts up-regulated by *Ascochyta* in chickpea suggests that the respective proteins may also have the capability to detoxify chickpea phytoalexins.

Complementing the detoxification of toxins, another fungal protection mechanism is the transport of the toxins out of the cell. Several transcripts up-regulated during infection may have this function as their corresponding proteins belonged to “ABC transporters” and “Major facilitator superfamily transporter” (MFS) families, both involved in the transport of drugs and other compounds. MFS are also involved in self-protection against own toxins and the secretion of these toxins into the host (Pitkin et al., 1996; Choquer et al., 2007), additional crucial processes for a successful pathogenesis.

### Production of toxins and other phytotoxic secondary metabolites

Besides their defense against plant toxins, necrotrophic fungi, such as *A. rabiei*, produce toxic compounds themselves to kill host cells. These include several secondary metabolites which may also exert a role in *A. rabiei* pathogenicity, as the functional category “secondary metabolic process” was over-represented in the subset of genes up-regulated during infection.

The solanapyrone-producing genes that we discussed already belong to this category though they are probably no virulence factor in *A. rabiei*, as mutants unable to produce the toxin do not show reduced virulence (Kim et al., 2013). Our results agree with this observation as none of the solanapyrone transcripts were differentially expressed during infection. By contrast, many transcripts putatively involved in toxin production were only expressed during infection (see Additional file 9 for a detailed description). Among these were five genes encoding “Nonribosomal peptide synthetases” (NRPSs) and two encoding “Polyketide synthases” (PKS). NRPSs are modular enzymes that synthesize a diverse set of secondary metabolites, including the dothideomycete host-specific toxins AM-toxin, HC-toxin, and victorin from *Alternaria* and *Cochliobolus* species (Hane et al., 2007). PKS are a family of proteins involved also in the synthesis of several mycotoxins and other secondary metabolites (Yun et al., 1998; Baker et al., 2006). Up to now only two *A. rabiei* genes encoding PKS, *pks1*, and *pks2*, were described (White and Chen, 2007; Akamatsu et al., 2010). We identified 12 proteins harboring the PKS domain in *A. rabiei* and two of their corresponding transcripts were up-regulated during infection, including *pks2*, but not *pks1*. In agreement with that, *pks1* was shown to be involved in 1,8-dihydroxynaphthalene-melanin pigment biosynthesis in *A. rabiei* and not needed for pathogenicity, while the *pks2* is in the same clade as *pksCT* which is responsible for biosynthesis of the nephrotoxic mycotoxin citrinin in *M. purpureus* (Akamatsu et al., 2010).

In addition to those mentioned above, a number of other genes only expressed during chickpea infection encoded proteins with homology to enzymes involved in toxin metabolism in other pathogens. Two of these predicted proteins had homology to *AFT1* and *AFT3* proteins involved in the synthesis of host-specific toxins in *Alternaria alternata* (Hatta et al., 2002), while others had homology with enzymes acting in aflatoxin and tricothecene metabolism in *A*. *parasiticus* and *F. sporotrichioides*, respectively. However, we could not identify unequivocal orthologs of these genes in *A. rabiei* since the percentage of identity between the predicted protein of *A. rabiei* genes and those of *A*. *parasiticus* and *F. sporotrichioides* was only moderate and mapping of these genes onto our draft *A. rabiei* genome did not reveal any clear cluster. Previous attempts based on reciprocal BLAST analyses to identify orthologs of aflatoxin synthesis-related genes in *Dothideomycetes* concluded that these fungi seemingly do not harbor orthologues of these genes. However, the abundance of non-reciprocal matches suggests the presence of paralogues for many of the genes (Ohm et al., 2012). In conclusion, we can not specify which toxins are produced by *A. rabiei* during infection, but the presence of genes with homology to toxin biosynthesis genes from other pathogens that were only expressed during host infection suggests that *A. rabiei* produces unknown toxins or secondary metabolites other than Solanapyrones and that these may be involved in pathogenicity. The identification of transcripts encoding potential toxin biosynthesis enzymes is an important result of our study and a relevant step to identify such toxins.

### Signaling during infection

Other pathogenesis-related genes up-regulated during infection include those involved in signaling. Cell-surface receptors perceive environmental signals that are transduced by G proteins into the cell and activate intracellular signaling pathways regulating i.a. host-pathogen interactions. We identified several transcripts of this signaling cascade putatively involved in infection-specific development. Thus, seven up-regulated transcripts encoded putative integral membrane proteins. Other up-regulated transcripts encoded putative members of the Rho family of G protein GTPases, or proteins acting in Rho protein activation or regulation. Cyclic AMP (cAMP) signaling, together with Mitogen-activated protein kinase (MAPK) cascades, play critical roles in the pathogenesis of several fungal pathogens (Yamauchi et al., 2004) and we found several up-regulated transcripts from this pathway.

### Conclusions

We here report the *de novo* assembly, annotation and characterization of the transcriptome of *A. rabiei* and predict the secretome of this devastating pathogen. By employing MACE as a novel, highly efficient tool for the analysis of interaction transcriptomes of eukaryotic hosts and pathogens we provide a comprehensive “*in planta*” inventory of *A. rabiei* genes consistently up-regulated or only expressed during host infection. These included several classes of candidate pathogenicity factors functioning in various aspects of infection and fungal defense processes. We feel that the work presented here is a first step toward the identification of crucial pathogenicity factors in *A. rabiei* and markedly improves our hitherto scarce understanding of the pathogen side of *Ascochyta*-legume pathosystems. Extension of the same type of analysis to other *A. rabei* pathotypes and *Ascochyta* species infecting other legumes will provide a firm fundament for the development of innovative, knowledge-based alternative strategies to control these important legume pathogens.

## Author contribution

SF carried out the inoculations with *A. rabiei*, extracted the RNA, performed the qRT-PCR assays, executed most of data analyses, participated in the design of the experiments and drafted the manuscript. BR developed MACE protocol, revised the manuscript and was involved in the design of the sequencing strategy. PW made substantial intellectual contributions to the writing of the manuscript. NK programmed and applied bioinformatic tools for analysis of MACE and RNA-seq, giving relevant advice for the data analysis strategy. GK supervised the research, improved the manuscript, conceived the study and participated in its design. All authors read and approved the final manuscript.

## Conflict of interest statement

The authors declare that the research was conducted in the absence of any commercial or financial relationships that could be construed as a potential conflict of interest.

## Supplementary material

### Availability of supporting data

This Transcriptome Shotgun Assembly project has been deposited at DDBJ/EMBL/GenBank under the accession GDJN00000000. The version described in this paper is the first version, GDJN01000000.

The raw MACE and RNA-Seq data supporting the result of this article are available in the Sequence Read Archive (SRA), with accession numbers SRR2086513-SRR2086543.

## References

[B1] Akamatsu H. O. Chilvers M. I. Kaiser W. J. Peever T. L. (2012). Karyotype polymorphism and chromosomal rearrangement in populations of the phytopathogenic fungus, *Ascochyta rabiei*. Fungal Biol. 116, 1119–1133. 10.1016/j.funbio.2012.07.001 23153803

[B2] Akamatsu H. O. Chilvers M. I. Stewart J. E. Peever T. L. (2010). Identification and function of a polyketide synthase gene responsible for 1,8-dihydroxynaphthalene-melanin pigment biosynthesis in *Ascochyta rabiei*. Curr. Genet. 56, 349–360. 10.1007/s00294-010-0306-2 20473673

[B3] Anders S. Huber W. (2010). Differential expression analysis for sequence count data. Genome Biol. 11:R106. 10.1186/gb-2010-11-10-r106 20979621 PMC3218662

[B4] Annis S. L. Goodwin P. H. (1997). Recent advances in the molecular genetics of plant cell wall-degrading enzymes produced by plant pathogenic fungi. Eur. J. Plant Pathol. 103, 1–14. 10.1023/A:1008656013255

[B5] Baker S. E. Kroken S. Inderbitzin P. Asvarak T. Li B. Y. Shi L.. (2006). Two polyketide synthase encoding genes are required for biosynthesis of the polyketide virulence factor, T-toxin, by *Cochliobolus heterostrophus*. Mol. Plant Microbe Interact. 19, 139–149. 10.1094/MPMI-19-0139 16529376

[B6] Bruns R. (1999). Molekularbiologische und Biochemische Untersuchungen zu Pektinolytischen Enzymen, Sowie zur Frage der Ploidie des Phytopathogenen Pilzes Ascochyta rabiei. Dissertation, Universität Münster, Münster, Germany.

[B7] Chen Y. M. Strange R. N. (1994). Production of a proteinaceous phytotoxin by *Ascochyta rabiei* grown in expressed chickpea sap. Plant Pathol. 43, 321–327. 10.1111/j.1365-3059.1994.tb02691.x

[B8] Choquer M. Lee M.-H. Bau H.-J. Chung K.-R. (2007). Deletion of a MFS transporter-like gene in *Cercospora nicotianae* reduces cercosporin toxin accumulation and fungal virulence. FEBS Lett. 581, 489–494. 10.1016/j.febslet.2007.01.011 17250832

[B9] Enkerli J. Bhatt G. Covert S. F. (1998). Maackiain detoxification contributes to the virulence of *Nectria haematococca* MP VI on chickpea. MPMI 11, 317–326. 10.1094/MPMI.1998.11.4.317

[B10] Ettinger W. F. Thukral S. K. Kolattukudy P. E. (1987). Structure of cutinase gene, cDNA, and the derived amino acid sequence from phytopathogenic fungi. Biochemistry 26, 7883–7892. 10.1021/bi00398a052

[B11] Finn R. D. Bateman A. Clements J. Coggill P. Eberhardt R. Y. Eddy S. R.. (2014). The Pfam protein families database. Nucleic Acids Res. 42, D222–D230. 10.1093/nar/gkt1223 24288371 PMC3965110

[B12] Grabherr M. G. Haas B. J. Yassour M. Levin J. Z. Thompson D. A. Amit I. (2011). Full-length transcriptome assembly from RNA-seq data without a reference genome. *Nat*. Biotechnol. 29, 644–652. 10.1038/nbt.1883 PMC3571712 21572440

[B13] Hane J. K. Lowe R. G. T. Solomon P. S. Tan K. C. Schoch C. L. Spatafora J. W.. (2007). Dothideomycete–Plant Interactions illuminated by genome sequencing and EST analysis of the wheat pathogen *Stagonospora nodorum*. Plant Cell 19, 3347–3368. 10.1105/tpc.107.052829 18024570 PMC2174895

[B14] Hatta R. Ito K. Hosaki Y. Tanaka Y. Tanaka A. Yamamoto M.. (2002). Conditionally dispensable chromosome controls host-specific pathogenicity in the fungal plant pathogen *Alternaria alternate*. Genetics 161, 59–70. 12019223 10.1093/genetics/161.1.59 PMC1462115

[B15] Hiremath P. J. Farmer A. Cannon S. B. Woodward J. Kudapa H. Tuteja R.. (2011). Large-scale transcriptome analysis in chickpea (*Cicer arietinum* L.), an orphan legume crop of the semi-arid tropics of Asia and Africa. Plant Biotechnol. J. 9, 922–931. 10.1111/j.1467-7652.2011.00625.x 21615673 PMC3437486

[B16] Höhl B. Weidemann C. Höhl U. Barz W. (1991). Isolation of the solanapyrones A, B and C from culture filtrates and spore germination fluids of *Ascochyta rabiei* and aspects of phytotoxin production. J. Phytopathol. 132, 193–206. 10.1111/j.1439-0434.1991.tb00112.x

[B17] Huang X. Madan A. (1999). CAP3: a DNA sequence assembly program. Genome Res. 9, 868–877. 10.1101/gr.9.9.868 10508846 PMC310812

[B18] Ilarslan H. Dolar F. S. (2002). Histological and ultrastructural changes in leaves and stems of resistant and susceptible chickpea cultivars to *Ascochyta rabiei*. J. Phytopathol. 150, 340–348. 10.1046/j.1439-0434.2002.00763.x

[B19] Imtiaz M. Abang M. M. Malhotra R. S. Ahmed S. Bayaa B. Udupa S. M. (2011). Pathotype IV, a new and highly virulent pathotype of *Didymella rabiei*, causing ascochyta blight in chickpea in Syria. Plant Dis. 95, 1192–1192. 10.1094/PDIS-04-11-0333 30732040

[B20] Jayakumar P. Gossen B. D. Warkentin T. D. Banniza S. (2005). Ascochyta blight of chickpea: infection and host resistance mechanisms. Can. J. Plant Pathol. 27, 499–509. 10.1080/07060660509507251

[B21] Kasahara K. Miyamoto T. Fujimoto T. Oguri H. Tokiwano T. Oikawa H.. (2010). Solanapyrone synthase, a possible diels–alderase and iterative type I polyketide synthase encoded in a biosynthetic gene cluster from *Alternaria solani*. Chem. Biochem. 11, 1245–1252. 10.1002/cbic.201000173 20486243

[B22] Kawahara Y. Oono Y. Kanamori H. Matsumoto T. Itoh T. Minami E. (2012). Simultaneous RNA-Seq analysis of a mixed transcriptome of rice and blast fungus interaction. PLoS ONE 7:e49423 10.1371/journal.pone.0049423 23139845 PMC3490861

[B23] Kessmann H. Barz W. (1986). Elicitation and suppression of phytoalexin and isoflavone accumulation in cotyledons of *Cicer arietinum* L. as caused by wounding and by polymeric components from the fungus *Ascochyta rabiei*. J. Phytopathol. 117, 321–335. 10.1111/j.1439-0434.1986.tb04370.x

[B24] Kim W. Akamatsu H. O. Peever T. L. Vandemark G. J. Chen W. (2013). Investigating the role of solanapyrone toxins in Ascochyta blight using toxin-deficient mutants of *Ascochyta rabiei*, Book of Abstracts, in *First Legume Society Conference: A Legume Odyssey* (Novi Sad), 203 (Accessed May 9–11, 2013).

[B25] Kraft B. Schwenen L. Stiickl D. Barz W. (1987). Degradation of the pterocarpan phytoalexin medicarpin by *Ascochyta rabiei*. Arch. Microbiol. 147, 201–206. 10.1007/BF00415285

[B26] Krogh A. Larsson B. von Heijne G. Sonnhammer E. L. L. (2001). Predicting transmembrane protein topology with a hidden markov model: application to complete genomes. J. Mol. Biol. 305, 567–580. 10.1006/jmbi.2000.4315 11152613

[B27] Latif Z. Strange R. N. Bilton J. Riazuddin S. (1993). Production of the phytotoxins, solanapyrones A and C and cytochalasin D among the nine isolates of *Ascochyta rabiei*. Plant Pathol. 42, 172–180. 10.1111/j.1365-3059.1993.tb01488.x

[B28] Li R. Li Y. Kristiansen K. Wang J. (2008). SOAP: short oligonucleotide alignment program. Bioinformatics 24, 713–714. 10.1093/bioinformatics/btn025 18227114

[B29] Lichtenzveig J. Winter P. Abbo S. Shtienberg D. Kaiser W. J. Kahl G. (2002). Towards the first linkage map of the *Didymella rabiei* genome. Phytoparasitica 30, 467–472. 10.1007/BF02979751

[B30] McCarthy F. M. Wang N. Magee G. B. Nanduri B. Lawrence M. L. Camon E. B.. (2006). AgBase: a functional genomics resource for agriculture. BMC Genomics 7:229. 10.1186/1471-2164-7-229 16961921 PMC1618847

[B31] Mistry J. Bateman A. Finn R. D. (2007). Predicting active site residue annotations in the Pfam database. BMC Bioinformatics 8:298. 10.1186/1471-2105-8-298 17688688 PMC2025603

[B32] Nagalakshmi U. Wang Z. Waern K. Shou C. Raha D. Gerstein M.. (2008). The transcriptional landscape of the yeast genome defined by RNA sequencing. Science 320, 1344–1349. 10.1126/science.1158441 18451266 PMC2951732

[B33] Nold-Petry C. A. Lo C. I. Rudloff I. Elgass K. D. Li S. Gantier M. P.. (2015). IL-37 requires the receptors IL-18Rα and IL-1R8 (SIGIRR) to carry out its multifaceted anti-inflammatory program upon innate signal transduction. Nat. Immunol. 16, 354–365. 10.1038/ni.3103 25729923

[B34] Ohm R. A. Feau N. Henrissat B. Schoch C. L. Horwitz B. A. Barry K. W.. (2012). Diverse lifestyles and strategies of plant pathogenesis encoded in the genomes of eighteen Dothideomycetes fungi. PLoS Pathog. 8:e1003037. 10.1371/journal.ppat.1003037 23236275 PMC3516569

[B35] Petersen T. N. Brunak S. von Heijne G. Nielsen H. (2011). SignalP 4.0: discriminating signal peptides from transmembrane regions. Nat. Methods 8, 785–786. 10.1038/nmeth.1701 21959131

[B36] Phan H. T. T. Ford R. Taylor P. W. J. (2003). Mapping the mating type locus of *Ascochyta rabiei*, the causal agent of ascochyta blight of chickpea. Mol. Plant Pathol. 4, 373–381. 10.1046/j.1364-3703.2003.00185.x 20569397

[B37] Pitkin J. W. Panaccione D. G. Walton J. D. (1996). A putative cyclic peptide efflux pump encoded by the *TOXA* gene of the plant-pathogenic fungus *Cochliobolus carbonurn*. Microbiology 142, 1557–1565. 8704997 10.1099/13500872-142-6-1557

[B38] Rouxel T. Grandaubert J. Hane J. K. Hoede C. van de Wouw A. P. Couloux A.. (2011). Effector diversification within compartments of the *Leptosphaeria maculans* genome affected by Repeat-Induced point mutations. Nat. Commun. 2, 202. 10.1038/ncomms1189 21326234 PMC3105345

[B39] Rubiales D. Fondevilla S. (2010). Resistance of cool season food legumes to ascochyta blight. Field Veg. Crop. Res. 47, 439–442. 10.3389/fpls.2012.00027 PMC3355812 22645577

[B40] Rubiales D. Fondevilla S. (2012). Future prospects for ascohyta blight resistance breeding in cool season food legumes. Front. Plant Sci. 3:27. 10.3389/fpls.2012.00027 22645577 PMC3355812

[B41] Schneider D. J. Collmer A. (2010). Studying plant-pathogen interactions in the genomics era: beyond molecular Koch's postulates to systems Biology. Annu. Rev. Phytopathol. 48, 457–479. 10.1146/annurev-phyto-073009-114411 20687834

[B42] Singh K. Nizam S. Sinha M. Verma P. K. (2012). Comparative transcriptome analysis of the necrotrophic fungus *Ascochyta rabiei* during oxidative stress: insight for fungal survival in the host plant. PLoS ONE 7:e33128. 10.1371/journal.pone.0033128 22427966 PMC3299738

[B43] Soanes D. M. Alam I. Cornell M. Wong H. M. Hedeler C. Paton N. W.. (2008). Comparative genome analysis of filamentous fungi reveals gene family expansions associated with fungal pathogenesis. PLoS ONE 3:e2300. 10.1371/journal.pone.0002300 18523684 PMC2409186

[B44] Tenhaken R. Arnemann M. Köhler G. Barz W. (1997). Characterization and cloning of cutinase from *Ascochyta rabiei*. Z. Naturforsch. 52c, 197–208. 10.1515/znc-1997-3-411 9167273

[B45] Tenhaken R. Barz W. (1991a). Characterization of pectic enzymes from the chickpea pathogen Ascochyta rabiei. Z. Naturforsch. 46c, 51–57.

[B46] Tenhaken R. Salmen H. Ch. Barz W. (1991b). Purification and characterization of pterocarpan hydroxylase, a flavoprotein monooxygenase from the fungus *Ascochyta rabiei* involved in pterocarpan phytoalexin metabolism. Arch. Microbiol. 155, 353–359. 10.1007/BF00243455

[B47] Thakur K. Chawla V. Bhatti S. Swarnkar M. K. Kaur J. Shankar R.. (2013). *De Novo* transcriptome sequencing and analysis for *Venturia inaequalis*, the devastating apple scab pathogen. PLoS ONE 8:e53937. 10.1371/journal.pone.0053937 23349770 PMC3547962

[B48] Trapnell C. Pachter L. Salzberg S. L. (2009). TopHat: discovering splice junctions with RNA-Seq. Bioinfirmatics 25, 1105–1111. 10.1093/bioinformatics/btp120 19289445 PMC2672628

[B49] White D. Chen W. (2007). Towards identifying pathogenic determinants of the chickpea pathogen *Ascochyta rabiei*. Eur. J. Plant Pathol. 119, 3–12. 10.1007/s10658-007-9122-z

[B50] Winnenburg R. Baldwin T. K. Urban M. Rawlings C. Köhler J. Hammond-Kosack K. E. (2006). PHI-base: a new database for pathogen host interactions. Nucleic Acids Res. 34, D459–D464. 10.1093/nar/gkj047 16381911 PMC1347410

[B51] Yamauchi J. Takayanagi N. Komeda K. Takano Y. Okuno T. (2004). cAMP-PKA signaling regulates multiple steps of fungal infection cooperatively with Cmk1 MAP Kinase in *Colletotrichum lagenarium*. MPMI 17, 1355–1365. 10.1094/MPMI.2004.17.12.1355 15597741

[B52] Yu J. Bhatnagar D. Cleveland T. E. (2004). Completed sequence of aflatoxin pathway gene cluster in *Aspergillus parasiticus*. FEBS Lett. 564, 126–130. 10.1016/S0014-5793(04)00327-8 15094053

[B53] Yun S. H. Turgeon B. G. Yoder O. C. (1998). REMI-induced mutants of *Mycosphaerella zeae*-*maydis* lacking the polyketide PMtoxin are deficient in pathogenesis to corn. Physiol. Mol. Plant Pathol. 52, 53–66.

[B54] Zawada A. M. Rogacev K. S. Müller S. Rotter B. Winter P. Fliser D.. (2014). Massive analysis of cDNA Ends (MACE) and miRNA expression profiling identifies proatherogenic pathways in chronic kidney disease. Epigenetics 9, 161–172. 10.4161/epi.26931 24184689 PMC3928179

