# Supplementary material for: In planta Identification of Putative Pathogenicity Factors from the Chickpea Pathogen Ascochyta rabiei by De novo Transcriptome Sequencing Using RNA-Seq and Massive Analysis of cDNA Ends
Source: Front Microbiol. 2015 Dec 1;6:1329. doi: 10.3389/fmicb.2015.01329 (PMC4664620; doi:10.3389/fmicb.2015.01329)
Supplement: Supplementary file 1 [file DataSheet1.DOCX]

**Hours after inoculation**

**%**

Additional file 1: Time course of *Ascochyta rabiei* infection cycle steps: spore germination, host’s epidermis penetration, development of necrotic lesions in the host’s mesophyll
