# Supplementary material for: In planta Identification of Putative Pathogenicity Factors from the Chickpea Pathogen Ascochyta rabiei by De novo Transcriptome Sequencing Using RNA-Seq and Massive Analysis of cDNA Ends
Source: Front Microbiol. 2015 Dec 1;6:1329. doi: 10.3389/fmicb.2015.01329 (PMC4664620; doi:10.3389/fmicb.2015.01329)
Supplement: Supplementary file 2 [file DataSheet2.DOC]

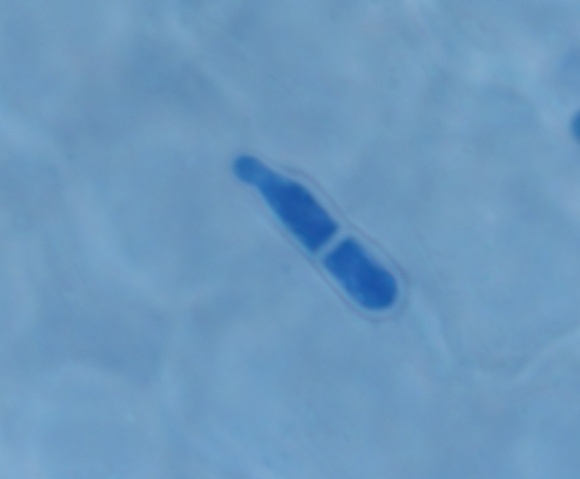

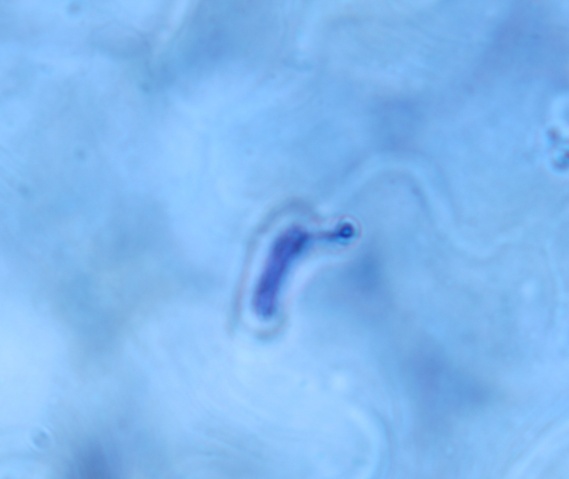


**A)**

**B)**


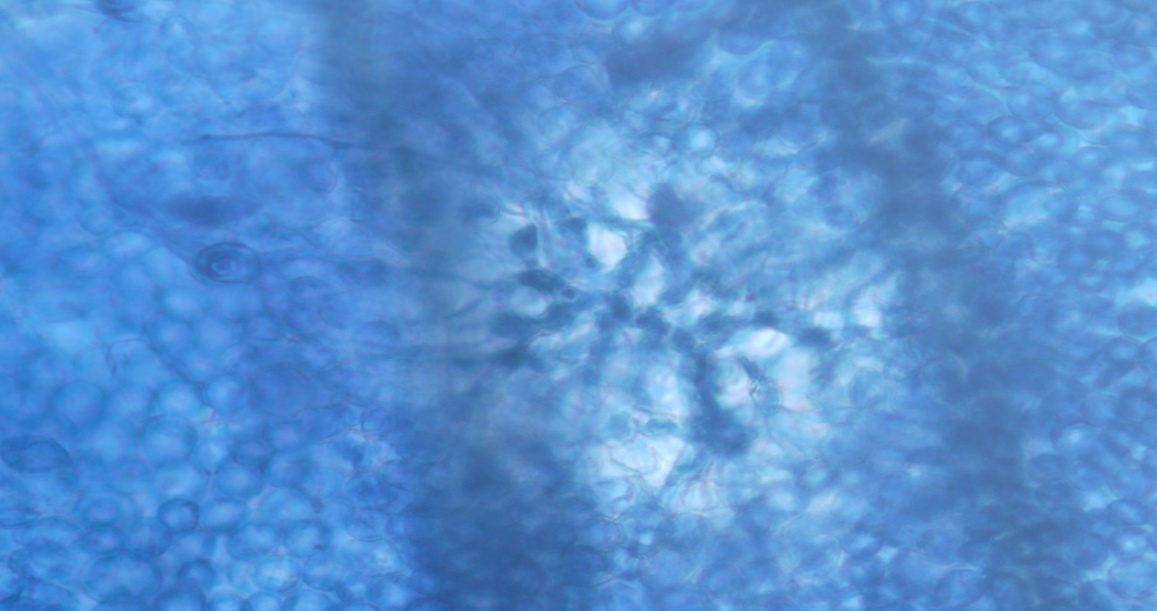


Additional file 2: Microscopic images illustrating *Ascochyta rabiei* developmental stages selected for sequencing. Observations were made using bright field. A) *A. rabiei* spore germinating 12hai. Germ tube is indicated by an arrow; B) *A. rabiei* spore penetrating a chickpea epidermal cell 36 hai. Penetration point is indicated by an arrow. C) Necrotic lesion caused by *A. rabiei* in chickpea mesophyll 96 hai.

**C)**
