# Supplementary figures and images for: In planta Identification of Putative Pathogenicity Factors from the Chickpea Pathogen Ascochyta rabiei by De novo Transcriptome Sequencing Using RNA-Seq and Massive Analysis of cDNA Ends
Source: Front Microbiol. 2015 Dec 1;6:1329. doi: 10.3389/fmicb.2015.01329 (PMC4664620; doi:10.3389/fmicb.2015.01329)

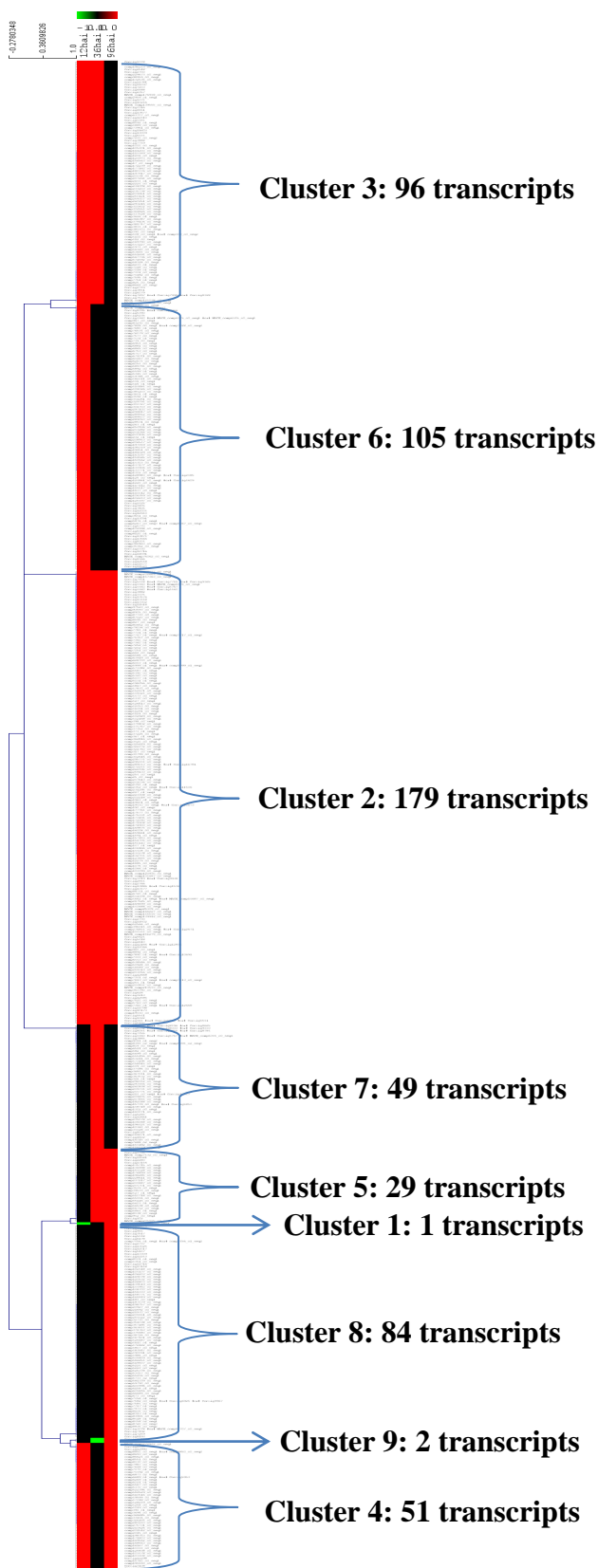

Supplement: Supplementary file 7 [file DataSheet7.PDF]
