# Supplementary material for: In planta Identification of Putative Pathogenicity Factors from the Chickpea Pathogen Ascochyta rabiei by De novo Transcriptome Sequencing Using RNA-Seq and Massive Analysis of cDNA Ends
Source: Front Microbiol. 2015 Dec 1;6:1329. doi: 10.3389/fmicb.2015.01329 (PMC4664620; doi:10.3389/fmicb.2015.01329)
Supplement: Supplementary file 9 [file DataSheet9.DOC]

**Additional file 9.** *A. rabiei* transcripts up-regulated in “*in planta*” compared to “*in medium*” treatments putative involved in the degradation of plant tissues, toxin metabolism and protection against fungitoxic compounds. A transcript was considered up-regulated when fold change ≥2 and p< 0.05 or when it was only identified in the “*in planta*” treatments.

| **ID** | **Annotation** | **Fold change (*in planta*/*in medium*)** | | |
| --- | --- | --- | --- | --- |
|  |  | **12 hai** | **36 hai** | **96 hai** |
| **Putative plant tissue degrading enzimes** | | | | |
| Contig13264 | Probable alpha-N-arabinofuranosidase b | nr* | nr | 171,9 |
| Comp74492_c0_seq1 | Tripeptidyl-peptidase 1 | *in planta* | absent | *in planta* |
| Contig1422 | Catechol 1,2-dioxygenase | *in planta* | *in planta* | *in planta* |
| Contig11497 | Lipase B | 99,46 | 112,29 | nr |
| Contig1097 | X-Pro dipeptidyl-peptidase C-terminal non-catalytic domain-containing protein | 129,70 | nr | 588,03 |
| Comp5876_c1_seq1 | Cholinesterase | 691,88 | nr | nr |
| Contig12940 | Cuticle-degrading protease | 2478,24 | nr | nr |
| Comp104344_c0_seq1 | Phospholipase D Active site motif protein | *in planta* | *in planta* | *in planta* |
| Comp276844_c0_seq1 #or# Contig2671 | Beta-glucosidase | *in planta* | *in planta* | *in planta* |
| Contig12676 | X-Pro dipeptidyl-peptidase protein | nr | nr | 168,85 |
| Comp541008_c0_seq1 | Probable exo-1,4-beta-xylosidase bxlB | *in planta* | *in planta* | *in planta* |
| Comp295004_c0_seq1 | Exo-1,4-beta-xylosidase xlnD | *in planta* | *in planta* | *in planta* |
| Comp42453_c0_seq1 | Cellobiose dehydrogenase | *in planta* | *in planta* | *in planta* |
| Comp427_c0_seq1 | Probable rhamnogalacturonate lyase A | *in planta* | *in planta* | *in planta* |
| Comp87740_c0_seq1 | Probable pectate lyase A | *in planta* | *in planta* | *in planta* |
| Comp106203_c0_seq1 | Carboxypeptidase S1 homolog B | absent | absent | *in planta* |
| Comp363644_c0_seq1 | Cutinase | absent | absent | *in planta* |
| Comp124973_c0_seq1 | Barwin-related endoglucanase | *in planta* | *in planta* | absent |
| Comp177160_c0_seq1 | Probable feruloyl esterase B-2 | *in planta* | *in planta* | absent |
| Comp51460_c0_seq1 | Cutinase | *in planta* | *in planta* | absent |
| Contig2749 | Galactan 1,3-beta-galactosidase | *in planta* | *in planta* | absent |
| Comp5699_c0_seq1 | Glucan 1,3-beta-glucosidase | absent | *in planta* | absent |
| Comp110012_c0_seq1 | Probable arabinan endo-1,5-alpha-L-arabinosidase A | *in planta* | absent | absent |
| Comp3592_c1_seq1 | Ligninase LG6 | *in planta* | absent | absent |
| Comp6054_c0_seq1 | Probable exopolygalacturonase B | *in planta* | absent | absent |
| Comp452971_c0_seq1 | Endo-1,3(4)-beta-glucanase 1 | *in planta* | *in planta* | *in planta* |
| **Transcripts putative involved in toxin metabolism** | | | | |
| Comp6443_c0_seq1 | Putative sterigmatocystin biosynthesis monooxygenase | *in planta* | *in planta* | *in planta* |
| Comp75357_c0_seq1 | Similar to beta-lactamase family protein | *in planta* | *in planta* | *in planta* |
| Contig2610 | Bifunctional solanapyrone synthase | *in planta* | *in planta* | *in planta* |
| Contig8366 | Trichodiene oxygenase | *in planta* | nr | nr |
| Contig8304 | Probable sterigmatocystin biosynthesis P450 monooxygenase | *in planta* | nr | nr |
| Contig2730 | Bifunctional solanapyrone synthase | *in planta* | *in planta* | *in planta* |
| Comp4662_c1_seq1 #or# MACE_comp14687_c0_seq1 | Nonribosomal peptide synthetase 14 | *in planta* | *in planta* | *in planta* |
| Comp25_c0_seq1 | FAD binding domain containing protein | *in planta* | *in planta* | *in planta* |
| Comp295774_c0_seq1 | Similar to polyketide synthase | *in planta* | *in planta* | *in planta* |
| Comp317_c0_seq1 | Isotrichodermin C-15 hydroxylase | *in planta* | *in planta* | *in planta* |
| Comp37352_c0_seq1 | Cytochrome P450 | *in planta* | *in planta* | *in planta* |
| Comp4121_c0_seq1 | Lovastatin nonaketide synthase | *in planta* | *in planta* | *in planta* |
| Comp56538_c0_seq1 | Isotrichodermin C-15 hydroxylase | absent | *in planta* | *in planta* |
| Comp103408_c0_seq1 | *A. rabiei* non-reduced type polyketide synthase protein (PKS2) gene | *in planta* | absent | *in planta* |
| Comp5370_c0_seq1 | Nonribosomal peptide synthetase 1 | *in planta* | absent | *in planta* |
| Comp161119_c0_seq1 | Nonribosomal peptide synthetase 14 | absent | absent | *in planta* |
| Comp184_c0_seq1 | Vacuolar membrane amino acid uptake transporter | absent | absent | *in planta* |
| Comp351038_c0_seq1 | Trichothecene 3-O-acetyltransferase | absent | absent | *in planta* |
| Comp375680_c0_seq1 | Putative acyl-coenzyme A synthetasa | absent | absent | *in planta* |
| Comp53457_c0_seq1 | L-pipecolate oxidase | absent | absent | *in planta* |
| Comp259307_c0_seq1 | Similar to putative non-ribosomal peptide synthase | *in planta* | *in planta* | absent |
| Contig7297 #or# Contig7998 #or# Contig8328 | Nonribosomal peptide synthetase | *in planta* | *in planta* | absent |
| Comp207845_c0_seq1 | Zinc-binding dehydrogenase family oxidoreductase | absent | *in planta* | absent |
| Comp326_c1_seq1 | Putative sterigmatocystin biosynthesis monooxygenase | absent | *in planta* | absent |
| Comp1137_c0_seq1 | Lovastatin nonaketide synthase | *in planta* | absent | absent |
| Comp134906_c0_seq1 | Lovastatin nonaketide synthase | *in planta* | absent | absent |
| Comp7680_c1_seq1 | O-methylsterigmatocystin oxidoreductase | *in planta* | absent | absent |
| Comp967_c0_seq1 | Nonribosomal peptide synthetase 13 | *in planta* | absent | absent |
| **Transcripts putative involved in protection against fungitoxic compounds** | | | | |
| Contig11798 | Pisatin demethylase | 145,46 | 382,32 | 932,76 |
| Comp21970_c0_seq1 | Similar to monooxygenase | absent | absent | *in planta* |
| Contig12661 | Aromatic peroxygenase | *nr* | 225,82 | nr |
| Comp7828_c0_seq1 | DNA repair and recombination protein rhm52 | *in planta* | absent | absent |
| Contig11477 | OsmC family protein | 58,85 | nr | nr |
| Contig11591 | Aromatic peroxygenase | 69,25 | nr | nr |
| Comp8120_c1_seq1 | Major facilitator superfamily transporter | 96,61 | nr | nr |
| Contig5147 | ABC transporter, putative | *nr* | nr | 447,08 |
| Contig2766 | MFS-type transporter C1271.10c | *in planta* | *in planta* | *in planta* |
| Comp4473_c0_seq1 | Rhodanese-like protein | *in planta* | *in planta* | *in planta* |
| Comp63919_c0_seq1 | Pisatin demethylase | *in planta* | *in planta* | *in planta* |
| Contig13448 | Drug/metabolite transporter | *in planta* | *in planta* | *in planta* |
| Comp368685_c0_seq1 | Aromatic peroxygenase | *in planta* | absent | *in planta* |
| Comp4019_c0_seq1 | Bypass of stop codon protein 6 | *in planta* | absent | *in planta* |
| Comp148700_c0_seq1 | mrr1 (mrr1) gene | absent | absent | *in planta* |
| Comp7584_c0_seq2 | Carboxylesterase | absent | absent | *in planta* |
| Comp249951_c0_seq1 | Similar to MFS transporter | *in planta* | *in planta* | absent |
| Contig7530 | Uncharacterized MFS-type transporter C18.02 | *in planta* | *in planta* | absent |
| Comp76079_c0_seq1 | Leptomycin B resistance protein pmd1 | *in planta* | absent | absent |

* nr= not differentially regulated; *in planta*= transcript identified only in the “*in planta”* treatment; absent= the transcript was not present at this time point neither “*in planta”*, nor “*in medium”* libraries
